# Elder abuse in the COVID-19 era based on calls to the National Center on Elder Abuse resource line

**DOI:** 10.1186/s12877-022-03385-w

**Published:** 2022-08-20

**Authors:** Gali H. Weissberger, Aaron C. Lim, Laura Mosqueda, Julie Schoen, Jenna Axelrod, Annie L. Nguyen, Kathleen H. Wilber, Richard S. Esquivel, S. Duke Han

**Affiliations:** 1 Interdisciplinary Department of Social Sciences, Bar-Ilan University, Ramat Gan, Israel; 2 Department of Family Medicine, USC Keck School of Medicine, 1000 S. Fremont Avenue, Unit 22, HSA Building A-6, Alhambra, CA 91803 USA; 3 USC Keck School of Medicine, 1975 Zonal Ave, Los Angeles, CA 90033 USA; 4 National Center On Elder Abuse, Department of Family Medicine and Geriatrics, 1000 S. Fremont Avenue, Unit 22, HSA Building A-6, Alhambra, CA 91803 USA; 5 USC Leonard Davis School of Gerontology, Los Angeles, CA 90089 USA; 6 NorthShore University HealthSystem, 909 Davis, Evanston, IL 60201 USA; 7 Rush Alzheimer’s Disease Center, Rush University Medical Center, 600 S. Paulina St, Chicago, IL 60612 USA; 8 Department of Psychology, USC Dornsife College of Letters, Arts, and Sciences, Los Angeles, CA 90089 USA; 9 Department of Neurology, USC Keck School of Medicine, Los Angeles, CA 90033 USA

**Keywords:** Elder abuse, COVID-19, Financial abuse, Emotional abuse, Physical abuse, Neglect, Family members

## Abstract

**Background:**

The COVID-19 pandemic has exacerbated circumstances that place older adults at higher risk for abuse, neglect, and exploitation. Identifying characteristics of elder abuse during COVID-19 is critically important. This study characterized and compared elder abuse patterns across two time periods, a one-year period during the pandemic, and a corresponding one-year period prior to the start of the pandemic.

**Methods:**

Contacts (including social media contacts, and email; all referred to as “calls” for expediency) made to the National Center on Elder Abuse (NCEA) resource line were examined for differences in types of reported elder abuse and characteristics of alleged perpetrators prior to the pandemic (Time 1; March 16, 2018 to March 15, 2019) and during the pandemic (Time 2; March 16, 2020 to March 15, 2021). Calls were examined for whether or not abuse was reported, the types of reported elder abuse, including financial, physical, sexual, emotional, and neglect, and characteristics of callers, victims, and alleged perpetrators. Chi-square tests of independence compared frequencies of elder abuse characteristics between time periods.

**Results:**

In Time 1, 1401 calls were received, of which 795 calls (56.7%) described abuse. In Time 2, 1009 calls were received, of which 550 calls (54.5%) described abuse. The difference between time periods in frequency of abuse to non-abuse calls was not significant ($p=0.28$). Time periods also did not significantly differ with regard to caller, victim, and perpetrator characteristics. Greater rates of physical abuse (${\upchi }^{2}=23.52, p<0.001)$ and emotional abuse (${\upchi }^{2}=7.12, p=0.008)$ were reported during Time 2 after adjustment for multiple comparisons. An increased frequency of multiple forms of abuse was also found in Time 2 compared to Time 1 (${\upchi }^{2}=23.52, p<0.001)$.

**Conclusions:**

Findings suggest differences in specific elder abuse subtypes and frequency of co-occurrence between subtypes between time periods, pointing to a potential increase in the severity of elder abuse during COVID-19.

**Supplementary Information:**

The online version contains supplementary material available at 10.1186/s12877-022-03385-w.

## Background

The COVID-19 pandemic has exacerbated circumstances that place older adults at higher risk for abuse, neglect, and exploitation. Consequences of elder abuse are grave, and result in negative physical, psychological, and social effects for victims, families/loved ones, communities, and society [1, 2]. In this study, we sought to characterize elder abuse patterns during a one-year period of the COVID-19 pandemic, and compare these patterns to a corresponding one-year period prior to the pandemic.

For several reasons, older adults may have been at increased risk of elder abuse during the height of the COVID-19 pandemic [3–6]. Limited interpersonal contact in order to prevent or slow virus transmission may lead to social isolation [5], a known risk factor for elder abuse [4, 7]. The pandemic may also increase the burden that caregivers experience and perceive in caring for older adults [5, 6, 8]. Moreover, older adults may be at higher risk for financial instability due to changes in money earning opportunities [5, 6], a factor linked to increased vulnerability to scams [9]. All of these factors may become particularly salient when concern of virus transmission grows and becomes widespread.

Few studies to our knowledge have examined rates and characteristics of elder abuse during the COVID-19 pandemic. While two survey studies found increased rates of elder abuse during the pandemic [10, 11] compared to a pre-pandemic period**,** one study [12] found evidence for decreased rates of elder abuse and age discrimination. A methodological limitation of the first two of these studies [10, 11] is that both utilized comparison datasets that were different from the pandemic dataset examined. More studies are needed to fully understand the scope of the impact of COVID-19 on elder abuse patterns.

In this study, we utilized contacts made to the National Center on Elder Abuse (NCEA) resource line to examine patterns of reported elder abuse over a one-year period after the United States federal government issued a stay-at-home order on March 16, 2020. We compared these patterns to patterns of reported elder abuse over a corresponding one-year period prior to the pandemic (March 16, 2018 to March 15, 2019). The NCEA resource line serves as a unique frontline source of data to investigate elder abuse characteristics across the United States [13]. Calls, emails, and social media messages to the NCEA during the two timeframes were descriptively examined for reports of elder abuse, the types of elder abuse described, and characteristics of the callers, the alleged perpetrators, and the victims. We expected that there would be an increase in elder abuse calls made to the NCEA resource line during the second time period, consistent with two recent studies [10, 11]. We also expected that there would be a shift in the distribution of reported abuse types and perpetrator characteristics across these two time periods. Given increased time at home and a decrease in access to home and community-based services during the second time period, we hypothesized that there would be a rise in reports of emotional and physical abuse in comparison to the prior time period. Due to increased economic vulnerability incurred by the pandemic, we predicted that a greater percentage of calls during the pandemic would report financial abuse. Additionally, we predicted that elder abuse during both time periods would be most commonly committed by a family member, consistent with previous work [13–17].

## Methods

### The National Center on Elder Abuse (NCEA)

The NCEA (https://ncea.acl.gov/) provides information and resources to individuals and community groups through multiple outlets, including a telephone line, website, and social media pages (all referred to as the NCEA resource line for purposes of this study). Individuals can contact the NCEA through these various outlets (for expediency, all forms of contact made to the NCEA resource line will be referred to as “calls” consistent with previously published work [13]. Calls made to the NCEA are summarized and logged into a database by NCEA staff. Responses to the calls by NCEA staff are also logged.

### Procedure

Study procedures were approved by the institutional review board of the University of Southern California. Calls made during the COVID-19 pandemic over a one-year period between March 16, 2020 and March 15, 2021 were coded (Time 2). To serve as a comparison, we also examined a one-year period of calls made prior to the pandemic from March 16, 2018 to March 15, 2019 (Time 1). Both years had an equal number of days (365).

A detailed description of the methodology for coding NCEA calls has been described in previous work [13]. In brief, prior to coding calls, an NCEA staff member de-identified the call narratives. Two independent raters then coded the calls with regard to whether or not abuse was reported, caller, victim, and perpetrator characteristics, the types of abuse alleged, whether multiple subtypes of abuse were alleged, and who perpetrated the alleged abuse. Call narratives and NCEA staff responses were utilized to code whether or not abuse was alleged. Single calls that reported two completely unique scenarios of abuse (two different victims or two different and unrelated perpetrators) were coded separately for each scenario of abuse and considered unique “calls” for analyses purposes. After identifying whether abuse was alleged, abuse calls were categorized into one or more of five elder abuse subtypes: financial, physical, sexual, emotional, and neglect. Calls were also coded for number of abusers reported per call (one abuser, more than one abuser, staff of a company or facility, or unable to determine) and the relationship of the abuser to the victim (family; non-family, non-medical caretaker; non-family, medical caretaker; caretaker, relationship unknown; an individual or entity known to the victim who does not fit the other categories; a stranger such as a telephone solicitor; or unable to determine). Two study co-authors (GHW, ACL) resolved any disagreements between the two independent raters.

Procedures for rating the calls followed the same codebook developed in previously published work [13]. The codebook was developed through a review of the scientific literature and expert knowledge on elder abuse. The two raters agreed on 85.35% of the initial codes (353 disagreements out of the 2410 total calls received) for overall alleged abuse prior to resolution of disagreements. Percent disagreement between raters on subtypes of abuse was calculated based on the number of times the raters disagreed about the subtype of abuse being reported out of the 1365 total calls reporting abuse across the two time periods. Disagreement was highest for financial and emotional abuse (11.2% and 11.8%, respectively), followed by neglect (9.01%), physical abuse (4.1%), and sexual abuse (0.4%). Non-abuse calls mostly consisted of general requests for information about the NCEA and elder abuse services.

Elder abuse and its subtypes were defined based on a Center for Disease Control and Prevention (CDC) report [18]. Per the CDC report, elder abuse is defined as “an intentional act or failure to act by a caregiver or another person in a relationship involving an expectation of trust that causes or creates a risk of harm to an older adult.” An older adult is defined as an individual 60 years of age or older. Consistent with previous work [13], we chose to include “strangers” when classifying abuser-victim relationships, a modification consistent with the U.S. Department of Justice’s Elder Justice Roadmap definition [19]. The general definition of elder abuse, definitions of specific subtypes applied in this study, and descriptions of relationships coded are described in detail in previous work [13] and in Supplemental Table 1.

### Additional criteria for coding abuse

Per CDC guidelines, alleged abuse between residents of long-term care facilities was not considered abuse. Calls reporting suboptimal living situations due to low income were not considered to be abuse for the purposes of this study.

Calls that reported abuse of an individual who is now deceased were only considered to be abuse if the death was presumed to be a result of the alleged abuse. This was done to ensure that abuse occurred within the two time periods of interest. Calls alleging abuse of victims residing outside of the United States or its territories and calls that alleged an abusive event that occurred prior to the windows of time under consideration were excluded from descriptive analyses. In the case of vague call narratives, abuse was only considered if the NCEA response narrative provided Adult Protective Services (APS) or police numbers to the caller or referenced a specific case of abuse.

### Analyses of calls

Total calls identifying alleged abuse for each time period were tallied and characteristics of the calls were summarized separately for each of the two time periods. Descriptive analyses procedures were as follows. If a call described two or more unique and unrelated instances of abuse, these instances were counted separately into the total. Percent of each abuse subtype was calculated by dividing the number of calls alleging a specific abuse subtype by the total number of calls reporting abuse. This same procedure was done to determine other characteristics of the calls, including caller, perpetrator, and victim characteristics, abuser-victim relationships and number of abusers. Calls that identified more than one subtype of abuse or relationship were included within each relevant descriptive analysis, such that some calls were represented more than once. In cases in which calls reported more than one subtype of abuse or other characteristic of interest (e.g., perpetrator relationship), the denominator remained the total number of calls reporting abuse, or the total number of calls reporting a subtype of abuse, in the case of subtype analyses (e.g., examining perpetrator relationships separately by subtype).

To investigate whether there were statistical differences in call characteristics between the two time periods, a series of chi-square tests of independence were conducted. Adjustments for multiple comparisons were made using Bonferroni corrections.

## Results

A total of 2410 calls were made across the two time periods. There were 1401 calls made during Time 1 (March 16, 2018 to March 15, 2019) and 1009 calls made during Time 2 (March 16, 2020 to March 15, 2021). Of the calls made during Time 1, 795 calls (56.7%) reported abuse of an older adult. During Time 2, 550 calls (54.5%) alleged abuse. There were 606 calls in Time 1 (43.3%) and 459 calls in Time 2 (45.5%) that did not allege abuse or were excluded from analyses (35 calls in Time 1, 2.5%; 36 calls in Time 2, 3.6%). The difference between time periods in frequency of abuse to non-abuse calls was not significant (${\upchi }^{2}=1.90, p=0.28$).

### Subtypes of alleged abuse

Table 1 presents the rates of abuse by subtype separately for Time 1 and Time 2, and results of the chi-square tests of independence. For both Time 1 and Time 2 financial abuse was the most commonly alleged abuse type, followed by emotional abuse, neglect, physical abuse, and sexual abuse.

**Table 1:** Frequency of abuse subtypes reported to the NCEA call center and results of chi-square tests of independence comparing frequencies of abuse subtypes between Time 1 and Time 2

	Time 1		Time 2		Chi-square results	
	Number	Percent	Number	Percent		*p*-value
Financial Abuse	364	45.79%	226	41.09%	2.911	*p* = 0.088
Physical Abuse	51	6.42%	79	14.36%	23.524	*p* < 0.001
Sexual Abuse	7	0.88%	8	1.45%	0.971	*p* = 0.324
Emotional Abuse	230	28.93%	197	35.82%	7.117	*p* = 0.008
Neglect	176	22.14%	117	21.27%	0.143	*p* = 0.705
Unspecified	142	17.86%	102	18.55%	0.184	*p* = 0.668
Mention of COVID-19	-	-	59	10.73%	-	-

*Note*: Time 1 represents abuse calls made between March 16, 2018 to March 15, 2019. Time 2 represents abuse calls made between March 16, 2020, to March 15, 2021. Sum of percentages may exceed 100% due to the fact that some calls alleged more than one subtype of abuse

**Fig. 1:**
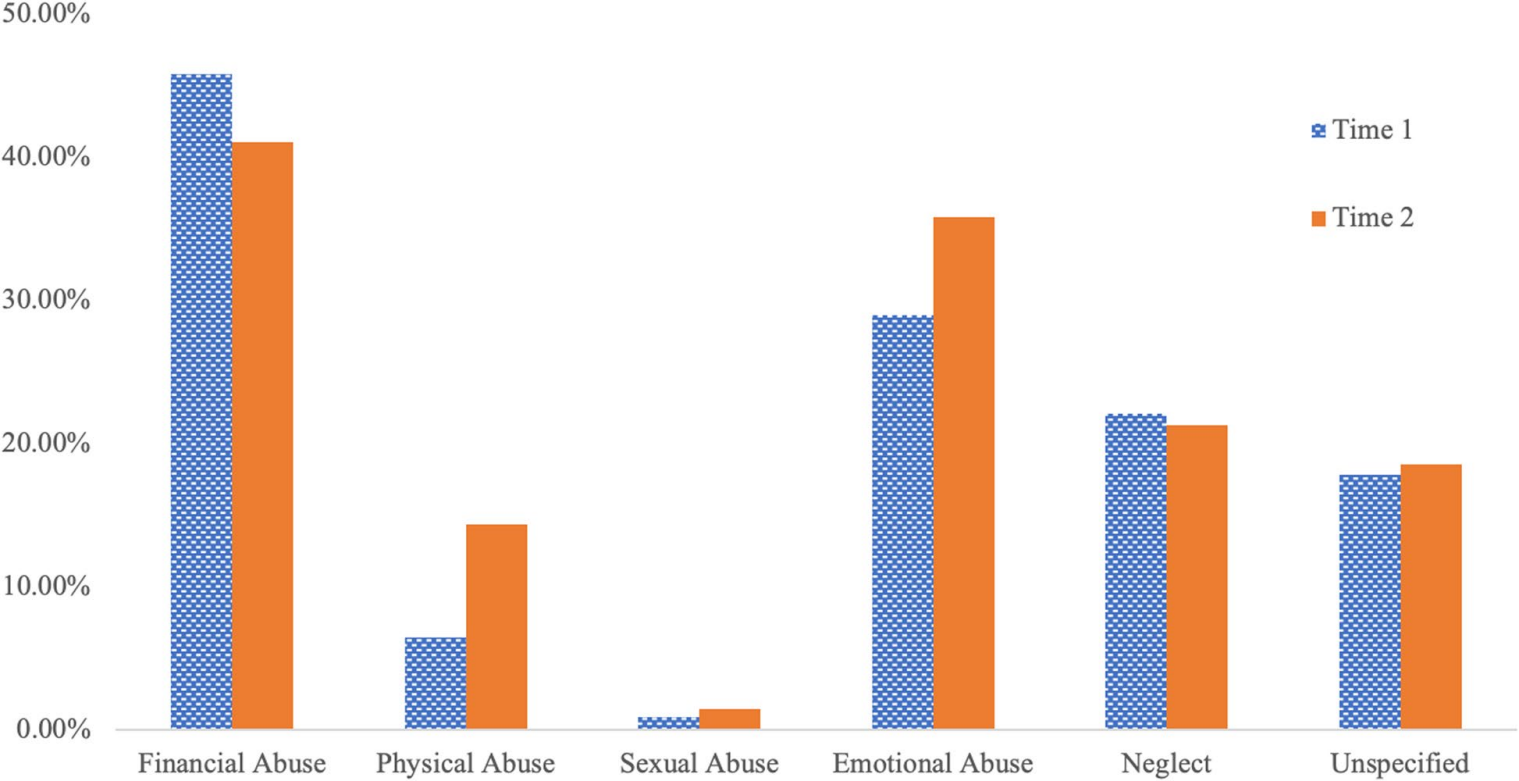
Frequency of abuse subtypes reported to the NCEA call center. Time 1 represents abuse calls (*n* = 795) made between March 16, 2018 to March 15, 2019. Time 2 represents abuse calls (*n* = 550) made between March 16, 2020, to March 15, 2021. Some calls reported more than one subtype of abuse, thus the percentages may exceed 100% for each time period

Comparing the frequency of the five abuse subtypes (financial abuse, physical abuse, sexual abuse, emotional abuse, and neglect) between Time 1 and Time 2 revealed a greater frequency of physical abuse calls (${\upchi }^{2}=23.52, p<0.001)$ and emotional abuse calls (${\upchi }^{2}=7.12 p=0.008)$ in Time 2 compared to Time 1. There were no significant differences in rates of alleged financial abuse (*p* = 0.09), sexual abuse (*p* = 0.32), and neglect (*p* = 0.71) between the two time periods. Frequencies of abuse subtypes between the two time periods can be viewed in Fig. 1.

### Caller, victim, and perpetrator characteristics

Calls were assessed for specific characteristics of the caller, the victim, and the perpetrator (Table 2). With regard to caller characteristics, in both time periods, most callers were not the victims themselves and this did not significantly differ between time periods (*p* = 0.15). Victims were most commonly reported as female in both time periods. With regard to sex of the alleged perpetrator, the vast majority of calls did not specify sex. Of the calls that specified sex, there were slightly more calls that reported female perpetrators for both time periods. There were no significant differences in sex breakdown between the two time periods (both *p*s ≥ 0.21).

**Table 2:** Caller, victim, and perpetrator characteristics

	Time 1 (795 calls)		Time 2 (550 calls)	
	Number	Percent	Number	Percent
Caller characteristics				
Self Report	183	23.02%	155	28.18%
Other Report	515	64.78%	362	65.82%
Unspecified	96	12.08%	33	6.00%
Victim Sex				
Male	171	21.51%	149	27.09%
Female	359	45.16%	263	47.82%
Unspecified	264	33.21%	138	25.09%
Perpetrator Sex^a^				
Male	121	15.22%	108	19.64%
Female	161	20.25%	116	21.09%
Unspecified	487	61.26%	312	56.73%
Number of Perpetrators				
One	370	46.54%	266	48.36%
More than one	91	11.45%	64	11.64%
Company or facility	152	19.12%	98	17.82%
Unable to determine	182	22.89%	122	22.18%
Relationship to Perpetrator^b^				
Family	270	33.96%	203	36.91%
Non-family, caretaker (medical)	138	17.36%	90	16.36%
Non-family, caretaker/guardian (non-medical)	14	1.76%	11	2.00%
Known, non-family, non-caretaker	140	17.61%	90	16.36%
Unknown (i.e., stranger)	60	7.55%	30	5.45%
Not reported	175	22.01%	118	21.45%
Caretaker relationship, unknown type	6	0.75%	8	1.45%

*Note*: Time 1 represents calls made between March 16, 2018 to March 15, 2019. Time 2 represents calls made between March 16, 2020, to March 15, 2021

^a^Sum of percentages may exceed 100% due to the fact that some calls alleged more than one perpetrator

^b^Sum of percentages may exceed 100% due to the fact that some calls alleged more than one relationship

The number of perpetrators discussed in each call was also assessed. Both time periods indicated one abuser for the majority of calls, followed by abuse by a company or facility, and more than one abuser. There were no significant differences in breakdown of number of abusers reported between the two time periods (*p* = 0.77).

**Fig. 2:**
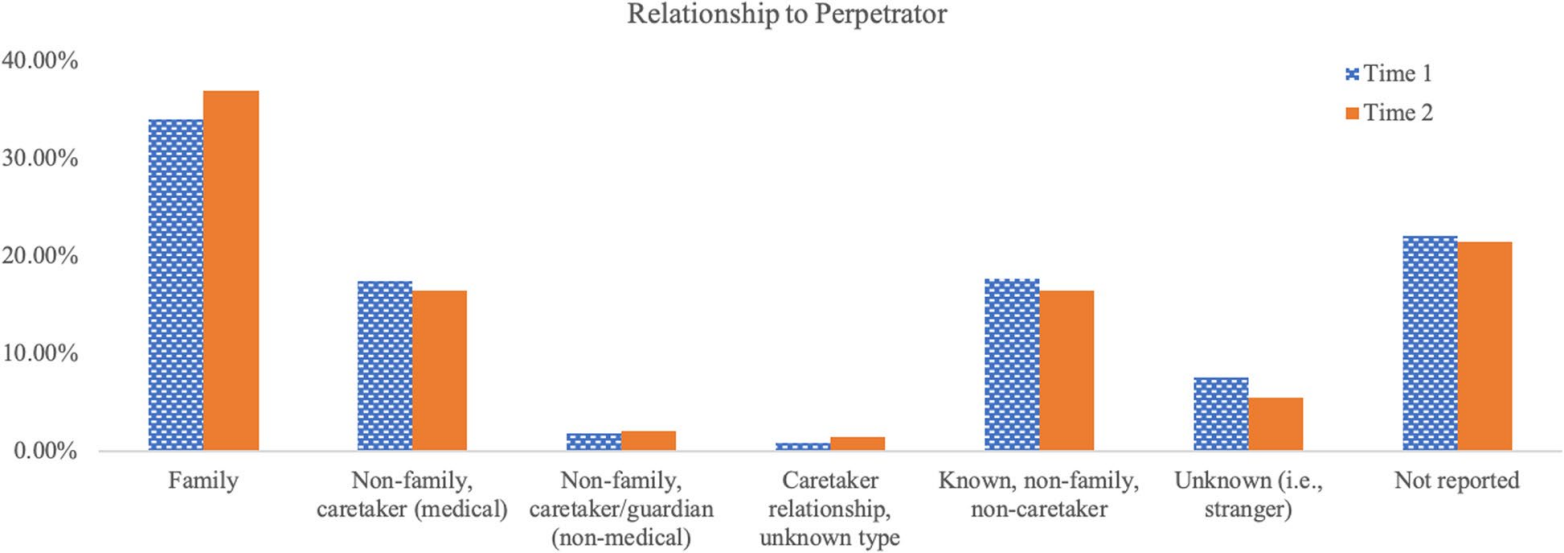
Breakdown of reported perpetrator’s relationship to the victim. Breakdown of reported perpetrator’s relationship to the victim separately for Time 1 (795 calls) and Time 2 (550 calls). Time 1 represents calls made between March 16, 2018 to March 15, 2019. Time 2 represents calls made between March 16, 2020, to March 15, 2021. Some calls reported more than one relationship, thus the percentages may exceed 100% for each time period

Calls were also assessed for the relationship reported between the perpetrator and the victim (see Table 2 and Fig. 2). For both time periods, family members were the most commonly alleged perpetrators, followed by relatively equal rates of calls reporting an individual known to the victim (non-family, non-caretaker) and non-family medical caretaker. Strangers were the next most common alleged perpetrators for both time periods, followed by non-family, non-medical caretaker, and unspecified caregivers. Differences did not arise with regard to the frequency of relationships reported across the two time periods (*p* = 0.36).

To further describe differences in the patterns of calls between the two time periods, we examined alleged victim-abuser relationships separately for the four most common abuse types between the two time periods: financial abuse, physical abuse, emotional abuse, and neglect (Supplemental Table 2 and Supplemental Fig. 1a-d). For both time periods, family members were the most commonly alleged perpetrators of financial, physical, and emotional abuse. For neglect, the most commonly alleged perpetrators for both time periods were medical caretakers. A pattern arose for physical abuse calls such that the percent of calls alleging a family member in Time 2 was lower by over 15% compared to Time 1, while the percent of calls alleging physical abuse by a non-family medical caretaker in Time 2 was higher by approximately 7% compared to Time 1.

Finally, we examined co-occurrence between abuse subtypes (Table 3). A significantly greater percentage of calls reported more than one abuse subtype in Time 2 (151 calls, 27.1%; ${\upchi }^{2}=23.52, p<0.001)$ compared to Time 1 (149 calls, 18.7%). Specific co-occurrences between subtypes are presented in Supplemental Table 3. For both time periods, financial abuse and physical abuse most commonly co-occurred with emotional abuse, and neglect most commonly occurred with financial abuse and emotional abuse.

**Table 3:** Number of calls that reported more than one abuse subtype for Time 1 and Time 2

	Time 1			Time 2		
	Number	Total Calls	%	Number	Total	%
Financial Abuse	113	364	31.0%	98	226	43.4%
Physical Abuse	32	51	62.7%	52	79	65.8%
Sexual Abuse	2	7	28.6%	7	8	87.5%
Emotional Abuse	115	229	50.2%	120	197	60.9%
Neglect	66	176	37.5%	55	117	47.0%
Total Across All Subtypes^a^	149	795	18.7%	151	558	27.5%

*Note*: The total number of calls reporting each subtype were used as each row’s denominator to calculate percentages. Note that row sums of Supplemental Table 3 (frequencies of co-occurrences between each subtype) differ from the corresponding total number of calls listed in Table 3 because some calls reported more than two different subtypes of abuse

^a^Statistically significant difference between Time 1 and Time 2 (${\upchi }^{2}=23.52, p<0.001)$

## Discussion

In this study, we examined calls made to the NCEA over a one-year period during the COVID-19 pandemic and compared them to calls made during a one-year period prior to the pandemic. Consistent with previous work by our group [13] and others [9, 20], during both time periods, financial abuse was the most commonly reported abuse subtype, followed by emotional abuse. Additionally, family members were the most commonly alleged perpetrators of abuse across both time periods [13–15, 17]. Other characteristics also did not differ between time periods including caller, perpetrator, and victim characteristics and number of perpetrators reported.

Differences between time periods arose when investigating frequencies of subtypes of abuse. Consistent with our prediction, a greater frequency of physical abuse and emotional abuse calls were reported in Time 2 compared to Time 1. This is consistent with a study that reported alarming increases in rates of domestic violence during the COVID-19 pandemic [21]. The authors [21] discuss that for individuals already in a vulnerable home situation, pandemic circumstances may exacerbate vulnerabilities. Older adults are more likely to be dependent on others for completion of daily activities due to physical and cognitive limitations that increase with age. All adults, including older adults, must be more reliant on technological forms of communication given physical distancing recommendations, and this greater dependency is increasingly being exploited by bad actors [22]. Greater dependencies on others and on technology can increase vulnerabilities of older adults during the pandemic, especially given increased pressures on caregivers and reduced access to outside supportive resources [4, 6].

The finding of increased physical and emotional abuse directly contrasts a study reporting a decrease in physical and psychological abuse during the pandemic compared to a pre-pandemic period in a representative community sample of older women in Hong Kong [12]. Our findings also diverge slightly from those of Chang et al. [10] who found increases in rates of physical abuse and financial abuse reported during the pandemic period, but not in verbal abuse (a type of emotional abuse). In their study, the authors compared results of an elder abuse survey administered during a two-week period during the COVID-19 pandemic to two nationally representative surveys conducted prior to the pandemic. Elder abuse subtypes were assessed using single item questions and were based on self-report. Thus, differences between this study and our findings may be due to differences in how elder abuse is measured and/or the specific data sources utilized (i.e., survey questions versus an elder abuse resource line). Differences between studies may also reflect the complexity of measuring elder abuse during the COVID-19 pandemic. For example, Yan et al. [12] discuss that the reduction in elder abuse found in their study may reflect a true reduction in elder abuse as a result of changing living situations, or may reflect a change in the willingness to report elder abuse during the pandemic period when more victims are trapped at home with perpetrators of violence. Thus, different data sources (i.e., survey, resource line) may yield vastly different results.

Contrary to our hypothesis and two previous studies [10, 11] that found an increase in elder abuse rates during the COVID-19 pandemic, we did not find an increase in elder abuse calls during the pandemic period (Time 2). Importantly, the two previous studies utilized data sources that diverged from their pre-pandemic comparison dataset, which may have contributed to differences in elder abuse rates for reasons other than pre-post pandemic changes. Although the difference in the ratio of abuse to non-abuse calls between pre-pandemic and pandemic time periods was not significant in our study, there was an overall decrease in contacts made to the NCEA during the pandemic period. It is possible that aspects of the pandemic may have affected individuals’ initiative to call the NCEA resource line to receive elder abuse related services and support. Consistent with this notion, a recent Adult Protective Services (APS) report found that many APS programs received fewer reports in the beginning of the pandemic [23]. One possibility for less reports during the pandemic is that COVID-19 preventative measures such as social distancing and isolation reduce social contact which may subsequently decrease the opportunities for abuse to be detected and reported [24]. This may be particularly relevant in older adults who are isolating with perpetrators of abuse, such as family members, as they may be controlling what is being seen or heard by others [24].

During both time periods, physical and emotional abuse were most likely to co-occur with other abuse subtypes. This finding is consistent with previous work [13, 25, 26]. We additionally found a greater proportion of calls alleging more than one abuse subtype in Time 2 compared to Time 1. This may suggest increased severity of abuse during the pandemic period, a possibility suggested by Makaroun et al. [5]. During the pandemic, many older adults may be sharing living arrangements with family members who may be home more often and more available due to changes in work schedules and shifts in social activities. Moreover, older adults may be spending significantly more time with family members or caretakers due to a lack of other supportive resources. Such drastic lifestyle changes may consequently increase mood disorders and substance use both in caregivers and older adults [5]. Furthermore, increased tensions brought on by reduced economic stability, shared living spaces, and fears/anxieties related to COVID-19 transmission may also be risk factors for increased frequency and severity of abuse [10], thereby increasing the likelihood that perpetrators commit additional forms of abuse (i.e., emotional abuse progressing to physical abuse).

This study has several limitations. Findings in this study are based on calls or messages made by individuals who contacted the NCEA resource line to receive information or seek advice about elder abuse. This self-selection bias may skew findings, and precludes determinations of elder abuse incidence and prevalence during the COVID-19 era. Relatedly, the COVID-19 pandemic may impact older adults’ contact with outside supportive systems that may assist in the detection of elder abuse. As such, any assessment of the degree of elder abuse during the COVID-19 pandemic may underestimate the issue. Finally, because investigation is not part of the NCEA resource line protocol, we were unable to substantiate the veracity of abuse claims made by callers, though there is no reason to believe calls were made disingenuously.

Nevertheless, findings of this study have important research and clinical implications. Future studies examining changes in elder abuse characteristics longitudinally and in concert with shifting social distancing patterns and virus transmission rates may further shed light on the complexities of the issue. Additionally, enhanced awareness (e.g., within healthcare organizations and amongst healthcare providers) of elder abuse risk factors such as social isolation, mental illness, and substance use that may change alongside evolving virus transmission rates and social distancing measures is critical [5, 6]. This will ultimately help identify those older adults most at risk and put in place protective measures so that abusive situations can be avoided.

## Conclusions

This is one of the only studies to compare elder abuse characteristics during the COVID-19 pandemic to a pre-pandemic period, and the only study to our knowledge to do so using the same data source for comparison. Findings suggest differences in specific elder abuse subtypes and frequency of co-occurrence between subtypes between time periods. Future studies are needed to investigate elder abuse characteristics in larger and more representative samples of older adults, and across different time periods of the pandemic, to further clarify the impact of the COVID-19 pandemic on patterns of elder abuse.

## Supplementary Information

**Additional file 1:** **Supplemental Table 1.** (a) Definitions used to code calls made to the NCEA helpline. Definitions are based on CDC guidelines with some modifications. (b) Descriptions of the types of relationships coded for. Adapted from Weissberger et al. [13]. **Supplemental Table 2.** Breakdown of perpetrator relationships to victim separately by the four most commonly reported abuse subtypes. Some calls reported more than one relationship, thus the percentages may exceed 100% for each time period. **Supplemental Table 3.** Number of calls that report co-occurring subtypes for Time 1 (Panel A) and Time 2 (Panel B). **Supplemental Figure 1a-d.** Visual display of breakdown of perpetrator relationships to victim separately by the four most commonly reported abuse subtypes: (a) financial abuse (b) emotional abuse (c) neglect and (d) physical abuse. Some calls reported more than one relationship, thus the percentages may exceed 100% for each time period.

## Data Availability

The datasets used and/or analyzed during the current study are not publicly available (dataset belongs to the National Center on Elder Abuse (NCEA)) but available from the NCEA on reasonable request.

## Abbreviations

NCEA: National Center on Elder Abuse
CDC: Center for Disease Control
APS: Adult Protective Services

## References

[1] Mosqueda L, Dong X (2011). Elder Abuse and Self-neglect:“I Don't Care Anything About Going to the Doctor, to Be Honest….”. JAMA.

[2] Pillemer K, Connolly M-T, Breckman R, Spreng N, Lachs MS (2015). Elder mistreatment: Priorities for consideration by the White House Conference on Aging. Gerontologist.

[3] Elman A, Breckman R, Clark S, Gottesman E, Rachmuth L, Reiff M (2020). Effects of the COVID-19 Outbreak on Elder Mistreatment and Response in New York City: Initial Lessons. J Appl Gerontol.

[4] Han SD, Mosqueda L (2020). Elder Abuse in the COVID-19 Era. J Am Geriatr Soc.

[5] Makaroun LK, Bachrach RL, Rosland A-M (2020). Elder abuse in the time of COVID-19—Increased risks for older adults and their caregivers. Am J Geriatr Psychiatry.

[6] Yunus RM, Abdullah NN, Firdaus MAM (2021). Elder abuse and neglect in the midst of COVID-19. J Glob Health.

[7] Burnes D, Pillemer K, Caccamise PL, Mason A, Henderson CR, Berman J (2015). Prevalence of and risk factors for elder abuse and neglect in the community: a population-based study. J Am Geriatr Soc.

[8] Schiamberg LB, Gans D (2000). Elder abuse by adult children: An applied ecological framework for understanding contextual risk factors and the intergenerational character of quality of life. Int J Aging Hum Dev.

[9] Acierno R, Hernandez MA, Amstadter AB, Resnick HS, Steve K, Muzzy W (2010). Prevalence and correlates of emotional, physical, sexual, and financial abuse and potential neglect in the United States: The National Elder Mistreatment Study. Am J Public Health.

[10] Chang E-S, Levy BR (2021). High prevalence of elder abuse during the COVID-19 pandemic: risk and resilience factors. Am J Geriatr Psychiatry.

[11] Du P, Chen Y (2021). Prevalence of elder abuse and victim-related risk factors during the COVID-19 pandemic in China. BMC Public Health.

[12] Yan E, Lai DW, Lee VW, Bai X, KL Ng H. Abuse and discrimination experienced by older women in the era of COVID-19: a two-wave representative community survey in Hong Kong. Violence Against Women. 2022;28(8):1750–72. 10.1177/10778012221085998 PMC9047603 35475662

[13] Weissberger GH, Goodman MC, Mosqueda L, Schoen J, Nguyen AL, Wilber KH (2019). Elder Abuse Characteristics Based on Calls to the National Center on Elder Abuse Resource Line. J Appl Gerontol.

[14] Biggs S, Manthorpe J, Tinker A, Doyle M, Erens B (2009). Mistreatment of older people in the United Kingdom: Findings from the first national prevalence study. J Elder Abuse Negl.

[15] Choi NG, Mayer J (2000). Elder abuse, neglect, and exploitation: Risk factors and prevention strategies. J Gerontol Soc Work.

[16] Lachs MS, Pillemer KA (2015). Elder abuse. N Engl J Med.

[17] Moon A, Lawson K, Carpiac M, Spaziano E (2006). Elder abuse and neglect among veterans in Greater Los Angeles: prevalence, types, and intervention outcomes. J Gerontol Soc Work.

[18] Hall, JE, Karch, DL, Crosby, AE. Elder Abuse Surveillance: Uniform Definitions and Recommended Core Data Elements For Use In Elder Abuse Surveillance, Version 1.0. Atlanta (GA): National Center for Injury Prevention and Control, Centers for Disease Control and Prevention; 2016.

[19] Connolly M, Brandl B, Brekman R. Elder justice roadmap report. Washington, DC: US Department of Justice Retrieved from; 2014. https://www.justice.gov/file/852856/download.

[20] Amstadter AB, Zajac K, Strachan M, Hernandez MA, Kilpatrick DG, Acierno R (2011). Prevalence and correlates of elder mistreatment in South Carolina: The South Carolina elder mistreatment study. J Interpers Violence.

[21] Boserup B, McKenney M, Elkbuli A (2020). Alarming trends in US domestic violence during the COVID-19 pandemic. Am J Emerg Med.

[22] Moore RC, Hancock JT. Older Adults, Social Technologies, and the Coronavirus Pandemic: Challenges, Strengths, and Strategies for Support. Social Media+ Society. 2020;6(3):1–5.

[23] Teaster P, Roberto K, Hoyt E, Savla J, Fua I, Kebede B (2020). Adult Protective Services Study on the Impact of COVID-19: Findings From State Administrator Survey and Interviews with Local APS Staff.

[24] Liu P-J, Delagrammatikas L (2021). Adult Protective Service's Role in Addressing Older and Dependent Adult Abuse in the Age of COVID. Front Public Health.

[25] Fisher BS, Regan SL (2006). The extent and frequency of abuse in the lives of older women and their relationship with health outcomes. Gerontologist.

[26] Post L, Page C, Conner T, Prokhorov A, Fang Y, Biroscak BJ (2010). Elder abuse in long-term care: Types, patterns, and risk factors. Res Aging.

