# Supplementary material for: Elder abuse in the COVID-19 era based on calls to the National Center on Elder Abuse resource line
Source: BMC Geriatr. 2022 Aug 20;22:689. doi: 10.1186/s12877-022-03385-w (PMC9392067; doi:10.1186/s12877-022-03385-w)
Supplement: Supplementary file 1 — Additional file 1: Supplemental Table 1. (a) Definitions used to code calls made to the NCEA helpline. Definitions are based on CDC guidelines with some modifications. (b) Descriptions of the types of relationships coded for. Adapted from Weissberger et al. [13]. Supplemental Table 2. Breakdown of perpetrator relationships to victim separately by the four most commonly reported abuse subtypes. Some calls reported more than one relationship, thus the percentages may exceed 100% for each time period. Supplemental Table 3. Number of calls that report co-occurring subtypes for Time 1 (Panel A) and Time 2 (Panel B). Supplemental Figure 1a-d. Visual display of breakdown of perpetrator relationships to victim separately by the four most commonly reported abuse subtypes: (a) financial abuse (b) emotional abuse (c) neglect and (d) physical abuse. Some calls reported more than one relationship, thus the percentages may exceed 100% for each time period. [file 12877_2022_3385_MOESM1_ESM.docx]

**Supplementary Legend**

**Supplemental Table 1.** (a) Definitions used to code calls made to the NCEA helpline. Definitions are based on CDC guidelines with some modifications. (b) Descriptions of the types of relationships coded for. Adapted from Weissberger et al.^11^

**Supplemental Table 2.** Breakdown of perpetrator relationships to victim separately by the four most commonly reported abuse subtypes. Some calls reported more than one relationship, thus the percentages may exceed 100% for each time period.

**Supplemental Table 3.** Number of calls that report co-occurring subtypes for Time 1 (Panel A) and Time 2 (Panel B).

**Supplemental Figure 1a-d. Visual display of breakdown of perpetrator relationships to victim separately by the four most commonly reported abuse subtypes: (a) financial abuse (b) emotional abuse (c) neglect and (d) physical abuse.** Some calls reported more than one relationship, thus the percentages may exceed 100% for each time period.

Supplemental Table 1. Definitions of elder abuse and elder abuse subtypes (A), and descriptions of reporter-abuser relationships (B).

| A. Definitions of abuse | | |
| --- | --- | --- |
|  |  |  |
| Abuse type | Definition^1^ | Examples |
| Elder Abuse^2^ | An intentional act or failure to act by a person in a relationship involving an expectation of trust (including self, acquaintances, or strangers) that causes or creates risk of harm to an older adult. |  |
| Physical Abuse | Intentional use of force resulting in physical pain, harm, injury, functional impairment, or distress. | Hitting, slapping, physical punishment, inappropriate use of medication or physical restraints. |
| Sexual Abuse | Forced or unwanted sexual contact or interaction. | Unwanted touching, fondling, suggestive talk, photography, voyeurism, or rape. |
| Emotional Abuse | Verbal or non-verbal interactions that result in fear, distress, psychological pain or anguish, or social or geographic isolation. | Threats (including physical), humiliation, disrespect. |
| Neglect | Failure by a caretaker to meet the basic needs (medical, nutrition, shelter, daily activities, protection) necessary for an older person’s physical and mental well-being. | Failure to provide adequate food, shelter, clothing, medical attention, or social stimulation. |
| Financial Exploitation^3^ | Improper, unauthorized, or fraudulent use of an older person’s property by a caregiver or trusted individual (abuse) or stranger (victimization) for the benefit of someone other than the elderly person. | Theft, inappropriate transfer or misappropriation of property; scams (sweepstake, grandparent, sweetheart) |
| Financial Abuse |  |  |
| Financial Victimization |  |  |
| B. Reported Abuser-Victim Relationship: | | |
| Relationship | Description |  |
| Family | Family member, including those designated as medical caretakers or with legal or fiduciary duties | |
| Non-family, medical caretaker | A person entrusted with the medical care or well-being of an older adult (e.g., doctors, nurses, nursing or home-health aides) | |
| Non-family, non-medical caretaker | A person entrusted with non-medical care or well-being of an older adult (e.g., legal guardian, POA, conservator) | |
| Known individual, not fitting above categories | An individual known to the victim but not fitting the above categories (e.g., friend, neighbor, lawyer (without guardian/conservator duties), financial manager, plumber etc). | |
| Stranger | An individual unknown to the victim (e.g., telephone or email scammer). | |
| Not reported | Relationship not mentioned in the “request narrative” or “response” descriptions | |

^1^ Definitions were adapted from CDC’s Uniform Definitions.

^2^The CDC definition of elder abuse was expanded to include abuse by strangers, in order to capture financial exploitation by scammers, and abuse by “self”, to include self-neglect. This is consistent with The Elder Justice Roadmap definition by the Departmet of Justice (2014).

^3^ Both financial abuse and victimization were considered financial exploitation in this study.

Supplemental Table 2. Breakdown of perpetrator relationships to victim separately by the four most commonly reported abuse subtypes.

|  | Financial Abuse | | | |  | Emotional Abuse | | | |  | Neglect | | | |  | Physical Abuse | | | |
| --- | --- | --- | --- | --- | --- | --- | --- | --- | --- | --- | --- | --- | --- | --- | --- | --- | --- | --- | --- |
|  | Time 1 | | Time 2 | |  | Time 1 | | Time 2 | |  | Time 1 | | Time 2 | |  | Time 1 | | Time 2 | |
|  | Calls | % | Calls | % |  | Calls | % | Calls | % |  | Calls | % | Calls | % |  | Calls | % | Calls | % |
| Family | 152 | 41.5% | 109 | 48.2% |  | 117 | 51.1% | 100 | 51.0% |  | 63 | 35.8% | 45 | 38.5% |  | 24 | 47.1% | 25 | 31.6% |
| Non-family, caretaker (medical) | 19 | 5.2% | 15 | 6.6% |  | 26 | 11.4% | 24 | 12.7% |  | 86 | 48.9% | 52 | 44.4% |  | 8 | 15.7% | 18 | 22.8% |
| Non-family, caretaker/guardian (non-medical) | 6 | 1.6% | 5 | 2.2% |  | 6 | 2.6% | 4 | 2.0% |  | 3 | 1.7% | 2 | 1.7% |  | 1 | 2.0% | 2 | 2.5% |
| Caregiver relationship, unknown type | 0 | 0.0% | 3 | 1.3% |  | 2 | 0.9% | 1 | 0.5% |  | 2 | 1.1% | 3 | 2.6% |  | 0 | 0.0% | 1 | 1.3% |
| Known, non-family, non-caretaker | 63 | 17.3% | 41 | 18.1% |  | 50 | 21.8% | 44 | 22.4% |  | 9 | 5.1% | 9 | 7.7% |  | 8 | 15.7% | 14 | 17.7% |
| Unknown (i.e., stranger) | 53 | 14.6% | 27 | 11.9% |  | 4 | 1.7% | 6 | 3.1% |  | 2 | 1.1% | 0 | 0.0% |  | 1 | 2.0% | 1 | 1.3% |
| Not reported | 69 | 19.0% | 26 | 11.5% |  | 21 | 9.1% | 17 | 8.7% |  | 11 | 6.3% | 6 | 5.1% |  | 9 | 17.6% | 18 | 22.8% |

Note: In few instances, calls reported more than one relationship. For simplicity, these are not included within frequencies presented in this table.

Supplemental Table 3. Number of calls that report co-occurring subtypes for Time 1 (Panel A) and Time 2 (Panel B).

|  | Time 1 | | | | | |  | Time 2 | | | | | |
| --- | --- | --- | --- | --- | --- | --- | --- | --- | --- | --- | --- | --- | --- |
|  | Financial Abuse | Physical Abuse | Sexual Abuse | Emotional Abuse | Neglect | Total Calls |  | Financial Abuse | Physical Abuse | Sexual Abuse | Emotional Abuse | Neglect | Total Calls |
| Financial Abuse | - | 17 | 1 | 81 | 39 | 364 |  | - | 18 | 0 | 71 | 32 | 226 |
| Physical Abuse | - | - | 1 | 19 | 11 | 51 |  | - | - | 3 | 38 | 10 | 79 |
| Sexual Abuse | - | - | - | 0 | 0 | 7 |  | - | - | - | 7 | 1 | 8 |
| Emotional Abuse | - | - | - | - | 39 | 229 |  | - | - | - | - | 30 | 197 |
| Neglect | - | - | - | - | - | 176 |  | - | - | - | - | - | 117 |

Note: FA = financial abuse; PA = physical abuse; SA = sexual abuse; EA = emotional abuse; Time 1 represents calls made between March 16, 2018 to March 15, 2019. Time 2 represents calls made between March 16, 2020, to March 15, 2021.

Supplemental Figure 1. Visual display of breakdown of perpetrator relationships to victim separately by the four most commonly reported abuse subtypes: (a) financial abuse (b) emotional abuse (c) neglect and (d) physical abuse.
